# Hæmoptysis—II

**Published:** 1910-07-16

**Authors:** Herbert French

**Affiliations:** Assistant Physician to Guy's Hospital.


### Hospital Clinics.

### HÆMOPTYSIS—II.

By HERBERT FRENCH, M.A., M.D. (Oxon.), F.R.C.P. (London.), Assistant Physician to Guy's Hospital.

Next to phthisis chronic valvular disease of the heart, especially mitral stenosis, is the commonest cause of hæmoptysis. The hæmoptysis in the case under discussion was finally diagnosed as due to cardiac disease. The cardiac lesion was probably not mitral stenosis, however, for the physical signs did not point to this, and the absence of a history of rheumatism, chorea, or scarlet fever was also against this view. The conclusion come to was that he was suffering from mitral regurgitation, the result of dilatation and hypertrophy of the left ventricle, the primary cause of which was chronic alcoholism. Post-mortem examinations not infrequently discover hypertrophy and dilatation of the heart with all the signs of backward pressure, without there being any disease of the valves, blood vessels, or kidneys, and in nearly all these cases there is a decided history or other good evidence of long-continued alcoholism. Excessive drinking as a definite cause of hypertrophy and dilatation of the heart is now recognised pretty generally.

### Hæmoptysis in Heart Disease.

The causes of hæmoptysis in heart disease will be best understood if one recalls the changes which take place in the lungs as a result of the backward pressure which is set up by stenosis or incompetence of the mitral valve.

1. The first result will be an obstruction of the flow of blood through the pulmonary veins, in consequence of which they themselves and their capillaries will become over-distended with blood (passive congestion of the lungs).

2. In addition there may be a transudation of serum and red blood corpuscles into the tissues of the air vesicles and into the air vesicles themselves, giving rise to hæmorrhagic œdema.

3. In more advanced cases the lung becomes tough, semi-solid, and airless, smooth on section, and dull red in colour. From its resemblance when cut to a section of the spleen this condition is called red splenisation or red induration. The change is due to a mixture of congestion of the pulmonary tissues—the arterioles of which are dilated, thickened, and atheromatous—œdema, actual hæmorrhage, and the fibrous organisation of former slight but repeated interstitial hæmorrhages.

4. In a still later stage the colour becomes brown from changes taking place in the blood pigment which has exuded into the connective tissues of the lung. That condition is called brown induration.

5. The lining membrane of the smaller bronchi may show signs of engorgement, and blood may exude from its surface.

6. Infarcts of various sizes may be found, dependent on the size of the vessels obstructed. The obstruction of the branch of the pulmonary artery involved may be the result either of embolism, or of thrombosis apart from embolism.

If due to embolism, it has been caused by the effect of the backward pressure which has produced stagnation of blood in the appendix of the right auricle, with consequent thrombosis there; the separation of a piece of this auricular clot furnishes the embolus. If due to a primary thrombosis, the clotting has been caused by the stagnation of the blood in the implicated vessel itself. The infarct is usually subpleural, firm in consistence, and of a dark brownish-red colour. The lung tissue becomes denucleated and necrotic, and the alveoli are filled with coagulated blood. The pleura over the infarct is usually inflamed. A considerable amount of dark blood may be expectorated when infarcts are present.

7. Another important change which is almost invariably found in these so-called cardiac lungs is thickening, dilatation, and atheromatous degeneration of the pulmonary arterioles, a condition brought about by the greatly increased tension produced in these vessels as a result of the backward pressure. Rupture of small branches of such degenerated pulmonary arteries also gives rise to hæmoptysis, and the alteration in the tunica intima, due to atheroma, together with the deficient rate of blood flow, strongly predisposes to thrombosis and infarction.

### In Injury and Pneumonia.

In cases of injury the lung is either wounded or ruptured, and the blood is derived from the severed branches of the pulmonary arterioles or veins. There is usually a history of, or evidence of, injury. There need be no cut or wound; a severe contusion or blow upon the thorax may cause hæmoptysis.

In pneumonia the amount of blood expectorated is in the majority of cases slight. The sputum is thick, viscid, tenacious, and generally of a russet-brown colour; it may, however, be bright red. At this stage there is inflammatory hyperæmia of the pulmonary capillaries and actual exudation of blood into the air vesicles. The disease is distinguished by the acute onset, the temperature, the high ratio of respiration rate to pulse rate, the characteristic viscidity of the sputa, and the presence of capsulated diplococci in the latter.

### In Acute Tuberculosis and Broncho-pneumonia.

In acute miliary tuberculosis the sputum may be tinged with blood, usually the result of inflammatory hyperæmia; seeing, however, that the disease affects infants and young children chiefly, and that at that age sputum is seldom expectorated, hæmoptysis is decidedly uncommon in acute general tuberculosis as distinct from acute phthisis.

In caseous broncho-pneumonia rapid breaking down of the lung tissue may occur, and free hæmoptysis may result from the rupture or erosion of branches of the pulmonary vessels. In a typical case one might find that the patient's illness began with rather free hæmoptysis and pyrexia, a diagnosis of incipient phthisis being made; a few days or weeks later signs of consolidation of the lung might develop in such a way as to suggest the possibility of lobar pneumonia. This view requires to be given up when it is found that the temperature still remains high at the end of a fortnight, and that the signs of consolidation are increasing, and the diagnosis is changed to that of tuberculous broncho-pneumonia, the patient rapidly wastes, becomes cyanosed, the temperature remains high with morning and evening fluctuations, and the physical signs increase in extent until death ensues a few weeks from the onset. In addition to extensive tuberculous broncho-pneumonia, evidence of older phthisis will generally be found in the form of local fibrosis and possibly deep-seated cavitation at one apex.

### Infarction.

Infarction of the lung has already been mentioned in connection with its most frequent cause, heart disease. It may also occur as a result of embolism secondary to thrombosis of the systemic veins, infective endocarditis of the pulmonary or tricuspid valves, and from primary thrombosis in some blood diseases such as leucocythæmia. A large infarct might give rise to pain, and on examination a local patch of dulness with bronchial breathing and a pleuritic rub might be detected. Hæmoptysis associated with such physical signs and accompanied by evidence of endocarditis or venous thrombosis would suggest infarct as the cause of it.

### New Growths.

Carcinoma and sarcoma of the lung are usually secondary. The primary seat of the growth may be near the lung: for instance, in the bronchus, œsophagus, breast, or mediastinal glands; or it may be distant, for instance, in the stomach, colon, and so on. The sputum may be tinged with blood, or it may be dark in colour and resemble in appearance red-currant jelly. Occasionally the hæmorrhage is profuse. Hæmoptysis may be due to hyperæmia and to breaking down of the growth, with ulceration or rupture of the branches of the pulmonary vessels, or the growth itself may be very vascular. A large number of cases of malignant growth in the lung are accompanied by pleuritic effusion, and growth may not at first be suspected. If aspiration is performed the fluid is generally found to contain blood; indeed, the discovery of blood-stained pleural fluid at a first tapping is always very suspicious of growth. Another important effect of growth is invasion of or pressure on a bronchus. The physical signs then are deficient movement, deficient or absent tactile vocal fremitus, dulness, deficient or absent breath sounds and vocal resonance over one lobe or one side of the chest, and accompanying these physical signs progressive weakness and emaciation, whilst no tubercle bacilli are found in the sputum. In at least one case of malignant growth of the lung presenting similar symptoms and physical signs to those just mentioned the malignant growth grew into a bronchus, forming a kind of cast of it which broke off and was expectorated. A microscopical examination showed it to be malignant in character, and although malignant disease of the lung had been diagnosed, it helped to confirm the diagnosis.

### Hydatids.

Hydatid cysts are rarer in Europe than they are in Australasia. Those of the lung are most commonly secondary to hydatid of the liver. Hæmoptysis is one of the most inportant symptoms; it may be the result of inflammatory hyperæmia or of actual destruction of lung tissue; the hydatid cysts may even cause compression of the lung and set up pneumonia or gangrene. The sputum may contain shreds of hydatid membrane, daughter cysts, scolices or hooklets. The physical signs are perhaps some local bulging of the thorax, dulness, deficient vesicular murmur, and vocal resonance. These signs may be associated with local enlargement of the liver. Dr. Taylor mentions in his book a case which was mistaken for phthisis. A girl with cerebral tumour in Guy's Hospital had hæmoptysis, and was thought to have tubercle of the brain and pulmonary phthisis, but a hydatid cyst was found in both brain and lung. The *x*-rays are very efficient in detecting the spherical shadows cast by hydatid cysts.

### Syphilis.

Syphilis may cause ulceration of the larynx, trachea, or bronchi, and hæmoptysis may result, blood vessels supplying these parts being involved and eroded. It may also, though rarely, give rise to the formation of gummata in the lungs. The gummata may soften, break down and form cavities, and hæmoptysis may result from the formation and rupture of pulmonary aneurysms, as in phthisis. The condition is most likely to be mistaken for phthisis. A history or evidence of syphilis and the absence of tubercle bacilli in the sputum would point to syphilis.

### Emphysema and Gangrene.

In emphysema the sputum may occasionally be streaked with blood which may be derived from a congested bronchial mucous membrane, or from the rupture of small capillaries in the walls of the air vesicles. The physical signs are sufficiently distinctive, but the sputum should be examined for tubercle bacilli with great care before one gives an opinion that hæmoptysis is due to emphysema or bronchitis only.

In gangrene of the lung there is a good deal of destruction of the lung tissue, in the course of which pulmonary vessels may be opened. It may be distinguished from most other diseases of the lung by the extremely fœtid and penetrating odour of the breath and by the sputum, which is also extremely fœtid, of a greenish-brown or dirty-grey colour. The fact that it contains fragments of necrosed lung tissue distinguishes it from the fœtid sputum that may be met with in some cases of bronchiectasis or of empyema ruptured into the lung.

### Ulceration and Abscess.

Single abscess of the lung is extremely rare. Multiple abscess may be found as a part of a general pyæmia. The sputum may be streaked with blood, the result of inflammatory hyperæmia.

Syphilitic, malignant, or tuberculous ulceration of the trachea or bronchi may cause hæmoptysis by erosion of branches of the bronchial vessels.

In bronchitis the sputum is occasionally streaked with blood which is derived from the congested blood vessels of the bronchial mucous membrane. The physical signs—namely, mucous râles and sibilant or sonorous rhonchi—which can be heard all over the chest are characteristic of this disease. Plastic bronchitis is distinguished by the characteristic sputum, casts of the bronchi being expectorated. It is essential, however, that the sputum should be examined carefully, and more than once for tubercle bacilli, lest the bronchitis is really masking incipient phthisis, the latter and not the bronchitis being the cause of the hæmoptysis.

### Bronchiectasis.

Bronchiectasis sometimes gives rise to profuse hæmoptysis, the patients being almost invariably admitted into the hospital as cases of phthisis. A careful physical examination and an examination of the sputum should enable one to make a correct diagnosis. Bronchiectasis, when it gives rise to hæmoptysis, is usually associated with fibroid change which is not tuberculous in origin. The physical signs of such a condition are: Flattening and deficient movement on the affected side, displacement of the cardiac impulse towards the affected side—*e.g.* when the right side is affected the cardiac impulse may be in the fifth right space in the right nipple line—increased tactile vocal fremitus, dulness, bronchial breathing, or even cavernous or amphoric breathing over a largely dilated tube, bronchophony, pectoriloquy, and loud consonating râles. With the exception of some compensatory emphysema, the other lung may show no evidence whatever of any morbid change. Another characteristic sign of bronchiectasis is the expectoration of a large quantity of sputum, half a pint, perhaps, or even more, in the early morning soon after the patient wakes and begins to move about. In phthisis, if one lung should give signs of such advanced disease, there would certainly be evidence of disease of the other lung also. In the post-mortem room one never sees the whole of one lung affected by phthisis and the other quite free. In fibroid lung and bronchiectasis, on the other hand, one not infrequently sees cases in which the whole of one lung is affected, the other being quite free.

### Aneurysmal Hæmoptysis.

An aneurysm of the aorta may open into the trachea or a bronchus; the hæmorrhage in such cases is usually profuse and rapidly fatal. Occasionally a slight leakage, causing moderate hæmoptysis, may first occur, and persist on and off even for a month or more before the fatal burst takes place. An aneurysm may also press on the lung and open into it, giving rise to hæmoptysis in this way. Before opening into the trachea or a bronchus an aneurysm usually first erodes the cartilages and then bulges into the lumen of the trachea or bronchus. It may often be diagnosed by a careful physical examination, attention being directed to the existence of a tumour, pain, pulsation, a bruit, and signs of pressure, such as inequality of the pulses, unequal pupils, recurrent laryngeal nerve paralysis, and tracheal tugging. The patient is generally a muscular man who had syphilis when young, who has worked hard physically, and who is not teetotal.

An œsophageal growth may cause hæmoptysis by invading the lung or treachea. It is usually preceded by dysphagia and rapid emaciation, and the diagnosis is not as a rule difficult. It has to be distinguished from œsophageal stenosis due to aneurysm of the descending thoracic aorta; the *x*-rays with and without bismuth soup may be of considerable assistance in arriving at the diagnosis.

A mediastinal growth may invade the lungs, trachea, or bronchi, and thus cause hæmoptysis. It is distinguished chiefly by its pressure effect on the superior vena cava, which gives rise to œdema of the arms, neck, and upper part of the chest, cyanosis of the face, and distension of the superficial veins of the chest wall.

### Hæmoptysis Due to Rare Causes.

Hæmoptysis may be caused by a parasite—the *distomum Wastermanni*—which invades the bronchial mucous membrane; however, it is not likely to be the cause of hæmoptysis in a patient who has not been in China, Japan, or Formosa.

Ulceration of the larynx as a cause for hæmoptysis is to be distinguished by there being signs of laryngeal disease, huskiness, aphonia, pain, etc., and by laryngoscopic examination.

Blood diseases may give rise to hæmoptysis just as they may to hæmorrhage from other parts. This may be due to changes in the vessels, or to an alteration in the condition of the blood, either of which conditions may lead to hæmorrhage. The history of the case, the general appearance of the patient, and a careful blood examination will generally suffice to make the diagnosis of hæmoptysis from any of these causes quite clear.

Vicarious menstruation is one of the conditions which is nearly always given in text-books as a cause of hæmorrhage from the lungs, nose, stomach, etc. It is of rare occurrence; it is even doubtful if hæmoptysis is ever really of this nature. When diagnosed, a lurking suspicion that it may really be due to early phthisis without physical signs must remain.

Hæmoptysis occasionally occurs in young, healthy subjects, and may disappear in a few days, never recur again, and leave no physical signs indicative of disease of the respiratory organs. Such cases should, however, be very carefully watched, as hæmoptysis coming on in this manner may be the first indication of phthisis.

